# RCSB Protein Data Bank: visualizing groups of experimentally determined PDB structures alongside computed structure models of proteins

**DOI:** 10.3389/fbinf.2023.1311287

**Published:** 2023-12-04

**Authors:** Joan Segura, Yana Rose, Chunxiao Bi, Jose Duarte, Stephen K. Burley, Sebastian Bittrich

**Affiliations:** ^1^ Research Collaboratory for Structural Bioinformatics Protein Data Bank, San Diego Supercomputer Center, University of California San Diego, San Diego, CA, United States; ^2^ Research Collaboratory for Structural Bioinformatics Protein Data Bank, Rutgers, The State University of New Jersey, Piscataway, NJ, United States; ^3^ Institute for Quantitative Biomedicine, Rutgers, The State University of New Jersey, Piscataway, NJ, United States; ^4^ Rutgers Cancer Institute of New Jersey, New Brunswick, NJ, United States; ^5^ Department of Chemistry and Chemical Biology, Rutgers, The State University of New Jersey, Piscataway, NJ, United States

**Keywords:** protein, structure, alignments, annotations, clustering, 3D visualization

## Abstract

Recent advances in Artificial Intelligence and Machine Learning (*e.g*., AlphaFold, RosettaFold, and ESMFold) enable prediction of three-dimensional (3D) protein structures from amino acid sequences alone at accuracies comparable to lower-resolution experimental methods. These tools have been employed to predict structures across entire proteomes and the results of large-scale metagenomic sequence studies, yielding an exponential increase in available biomolecular 3D structural information. Given the enormous volume of this newly computed biostructure data, there is an urgent need for robust tools to manage, search, cluster, and visualize large collections of structures. Equally important is the capability to efficiently summarize and visualize metadata, biological/biochemical annotations, and structural features, particularly when working with vast numbers of protein structures of both experimental origin from the Protein Data Bank (PDB) and computationally-predicted models. Moreover, researchers require advanced visualization techniques that support interactive exploration of multiple sequences and structural alignments. This paper introduces a suite of tools provided on the RCSB PDB research-focused web portal RCSB. org, tailor-made for efficient management, search, organization, and visualization of this burgeoning corpus of 3D macromolecular structure data.

## 1 Introduction

Recent developments in sequence-based protein structure prediction, which involve predicting the 3D structure of a protein based solely on its amino acid sequence, have revolutionized the way in which biostructure data are generated. New Artificial Intelligence/Machine Learning (AI/ML) methods based on deep learning (Jumper et al., 2021; Baek et al., 2021; Lin et al., 2023) are now capable of predicting 3D structures (or generating Computed Structure Models, CSMs) of proteins comparable in accuracy to lower-resolution experimentally-determined structures. This family of methods has increased the volume of available 3D structural information by three orders of magnitude *versus* the archival contents of the Protein Data Bank (PDB) (Crystallography, 1971). Various database (DB) resources compiling predicted structures of proteins have emerged from AI/ML approaches. AlphaFold Protein Structure DB (Varadi et al., 2022), in its current version, stores predicted structures for more than 214 million sequences from the universal Protein Resource (UniProt; UniProt Consortium, 2023) that account for nearly all proteins known to science. The ModelArchive is a repository designed specifically for storing and sharing computational models in a standardized format (Schwede et al., 2009). Currently, more than 74,000 models predicted using various methods, including AlphaFold2 and RosettaFold are available in the archive. The ESM Atlas (esmatlas.com) encompasses an enormous collection of more than 617 million metagenomic protein structure models, computed using ESMFold (Lin et al., 2023). The future of AM/ML technologies applied to protein structure prediction looks even more promising. Several studies have shown that AlphaFold2 can be used to predict structures of macromolecular assemblies (*e.g*., Evans et al., 2022). Other recent innovations have utilized similar AI/ML technologies to tackle the problem of predicting 3D structures of protein-ligand complexes (Hekkelman et al., 2023).

CSMs have also opened up entirely new avenues for studying the structural space of proteins at the organism proteome level and varying levels of detail, ranging from single-domain proteins to large macromolecular assemblies (*e.g*., Zhao et al., 2022; Koehler Leman et al., 2023; Durairaj et al., 2023). While experimental structures deposited into the global PDB archive covered only ∼17% of the human proteome’s amino acid residues, inclusion of CSMs from AI/ML approaches increases structural coverage of the human proteome to ∼58% of amino acid residues currently defined by experimental methods or confidently predicted by AI/ML software (Varadi et al., 2022). A similar trend is observed at a larger scale: CSMs have greatly expanded the number of organisms for which 3D structural information is available from 10,000 to more than 119,000 organism proteomes defined in UniProt. Working with 3D structure information at this scale, however, requires efficient tools for search, clustering, and visualization. Moreover, leveraging 3D information for large collections of protein structures including both experimentally-determined PDB structures and CSMs requires new large-scale approaches to summarize and display metadata and related biological/biochemical annotations of the proteins and their structural features, together with effective methods for visualizing multiple sequence and structure alignments interactively.

In 2022, the Research Collaboratory for Structural Bioinformatics Protein Data Bank (RCSB PDB) incorporated CSMs of more than one million proteins into its research-focused web portal, RCSB.org (Bittrich et al., 2023; Burley et al., 2023). Incorporation of CSMs with experimentally-determined PDB structures provides users with a much more comprehensive view of structural proteomes at the organism level with greater coverage of amino acid residues. It also enables utilization of RCSB.org tools for unified analysis of 3D biostructures, including searching, clustering, and visualizing experimentally-determined structures alongside CSMs (Burley et al., 2023). At present, RCSB.org supports simultaneous access to more than one million publicly-available CSMs from AlphaFold DB (alphafold.ebi.ac.uk) and the ModelArchive (modelarchive.org), together with more than 210,000 experimentally-determined structures housed in the continuously growing PDB archive. Incorporation of CSMs at RCSB.org empowers users to harness the RCSB.org advanced search functionality to construct intricate queries that meld database features with scientific searches (*e.g*., sequence, structure, and chemical similarity searches), facilitating exploration of tailor-made subsets of protein structures, including both experimentally-determined structures and CSMs. Furthermore, users can group search outcomes under various parameters and delve into distinct clusters of protein structures. “Groups” are defined as sets of proteins that share similarities in some way, shape, or form. For example, amino acid sequence-based similarity or proteins that share the same UniProt accession code or ID. Grouping of search results aids in filtering structural redundancy and provides an efficient approach to exploring the diversity of protein structures returned by a search. In addition, clustering large collections of protein structures into Groups facilitates identification of shared annotations among similar proteins and allows users to infer structural and biological/biochemical features from well-studied PDB structures and attribute or transfer them to CSMs. The RCSB.org web portal is equipped with tools that present summaries of biological/biochemical attributes and 3D structure metadata for grouped proteins and supports interactive alignment and visualization of Group member sequences and 3D structures.

In this manuscript, we explore diverse methodologies, workflows, and tactics employed by RCSB.org for searching, clustering, and visualization of ensembles of proteins in “Groups.” We enumerate the technologies and libraries harnessed in developing new visualization tools, together with software and algorithms employed for clustering more than one million 3D biostructures - both experimentally-determined PDB structures and CSMs. Additionally, we discuss the approach that RCSB.org employs for on-the-fly clustering of protein structures derived from search results. To conclude, we present several use cases exemplifying how analysis of protein Groups can aid in understanding protein structure and function.

## 2 Methods

### 2.1 Structures in RCSB.org

Macromolecular structure data follow a hierarchical organization in nature. For instance, macromolecular assemblies can be comprised of one or more protein subunits and small molecule ligands that form a stable 3D conformation. RCSB.org follows the structural hierarchy defined by PDB to organize 3D biostructures. The most important hierarchical terms used in this work are.

1. PDB Entry constitutes a unique record within the structural archive that describes the data and metadata of a 3D biostructure deposited by an author.
2. PDB Entity represents a unique molecular component within a PDB Entry. PDB Entities can include polymeric or small molecule ligands. Polymeric PDB Entities are differentiated by their sequence and chemical composition.
3. PDB Instance describes a particular occurrence of a PDB Entity within a PDB Entry. For instance, a homodimer can be composed of two occurrences of a single PDB Entity.
4. PDB Assembly defines the arrangement and organization of one or more PDB Instances that form a biologically functional unit within a PDB Entry.

Structure Summary Pages (SSP) serve as a centralized hub to explore PDB Entries or structures and their different parts (PDB Entities, PDB Instances, and PDB Assemblies). These pages amalgamate a wealth of data and provide access to different computational tools, offering an in-depth portrayal of the deposited structure. SSP provides essential information such as the experimental method, resolution metrics, biological context, release date, and publication. In addition, quality metrics and related structural data are also presented, enabling a comprehensive assessment of the structure’s reliability and biological relevance. The SSP gives access to interactive visualizations of the 3D structure and sequence positional annotations. The inclusion of internal and external links to related specialized analytical tools and databases further enriches PDB Entries, allowing users to find other related biostructures and relevant biological information. Similarly to experimentally-determined PDB Entries, CSM data and metadata can be accessed through SSPs. In those cases, experimental-related quality metrics and metadata are replaced by a distribution chart of the per-residue prediction confidence score (pLDDT) (Jumper et al., 2021) and information on the CSM provenance.

### 2.2 Groups in RCSB.org

While SSPs provide a detailed snapshot of crucial information pertaining to individual structures, there are many research settings in which analyses across multiple proteins can reveal patterns in both 1D sequences and 3D structures that might be overlooked when analyzing proteins one at a time. RCSB.org offers a comprehensive suite of tools designed to analyze and explore Groups of protein structures clustered by distinct attributes or features, including clusters of proteins defined by sequence identity, proteins bearing the same UniProt ID, or ensembles of structures that have been deposited together by researchers. Our tools designed for analyzing Groups permit a much deeper understanding of structural and functional annotations across multiple proteins highlighting differences among their 1D sequences (*e.g.*, amino acid changes) and 3D structures (*e.g.*, conformational changes). Since Groups can contain both experimentally-determined structures and CSMs, Group tools enable efficient comparisons between different types of 3D biostructure information.

Currently, RCSB.org employs three primary methods to assigning protein structures to Groups.

1. Deposition Groups: These Groups encompass structures that researchers deposit simultaneously via the GroupDep tool (Burley et al., 2019). Such collections of typical structures explain how the same protein interacts with a variety of ligands, drugs, or drug candidates. Metadata items such as sample pH and temperature, reflecting experimental conditions, might also be used to determine Group membership.
2. UniProt Groups: These Groups gather all protein sequences represented in the PDB or CSMs incorporated into RCSB.org that share the same UniProt ID. Connecting PDB structures and UniProt IDs relies on the SIFTS resource (Dana et al., 2019). Metadata associated with all CSMs incorporated at RCSB.org includes UniProt IDs.
3. Sequence Identity Groups: These Groups are defined by calculating sequence identity clusters of amino acid residue sequences across both the PDB archive and the CSMs incorporated at RCSB.org. Clusters are updated weekly to include newly-released PDB structures and newly-incorporated CSMs using the MMSeqs2 software suite (Steinegger and Söding, 2017). Multiple clustering results are made available for various sequence similarity thresholds.

RCSB.org grouping strategies function at different levels of granularity. Deposition Groups consist of structures that were submitted together. They are made up entirely of PDB structures, whether composed of 1 or more polymeric molecules (protein or nucleic acid chains). Conversely, both UniProt and Sequence Identity Groups encompass individual protein sequences corresponding to polymeric PDB Entities (unique polymer chains differentiated by their sequence and chemical composition within a PDB structure) or incorporated CSMs.

**FIGURE 1:**
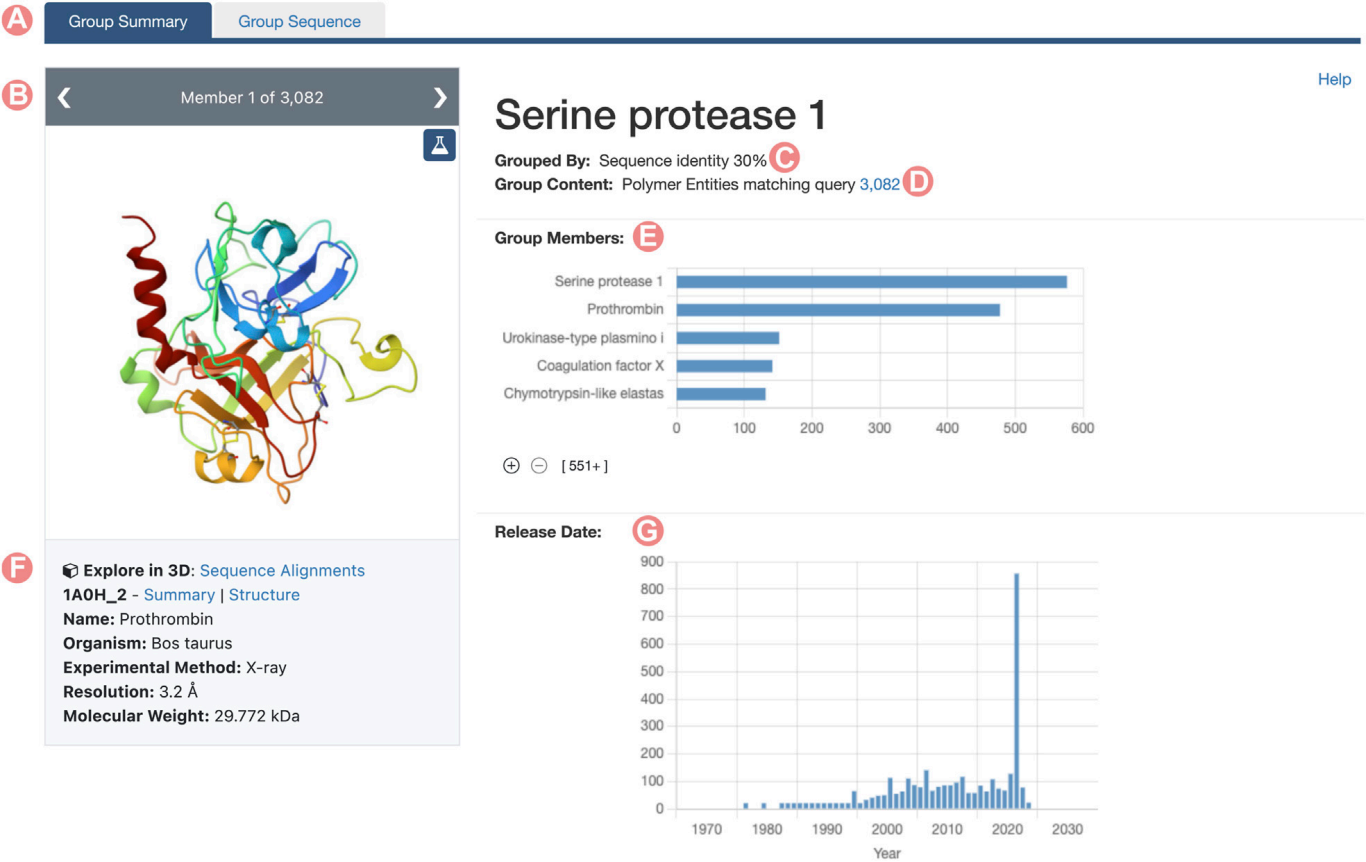
Serine protease Sequence Identity Group Summary Page header. Group Summary Pages (GSPs) are the main landing pages for RCSB PDB groups of proteins. The GSP header includes **(A)** Tab to navigate between GSP and Group Sequence Page (Contains sequence information of the group proteins). **(B)** The slide deck component displays the Group members. **(C)** Group aggregation method. **(D)** Number of elements included in the Group. **(E)** Distribution of Group member names. **(F)** Information and links for the Group members are displayed in the slide deck. **(G)** Histogram of release dates for Group members. The 2021 histogram peak includes the incorporated CSMs on the RCSB.org portal.

Similarly to SSPs, RCSB.org Group Summary Pages (GSPs) serve as the primary entry point to explore Groups of PDB structures and/or CSMs. These pages consolidate information about the Group and the characteristics of its members with a layout that resembles the RCSB.org Structure Summary Page. The header of each GSP features the Group title, the methodology used for grouping members, and the total count of elements within the Group (Figures 1C, D). It also includes links to RCSB.org Advanced Search capabilities, showcasing the Group’s content (PDB structures for Deposition Groups and polymeric PDB Entities and CSMs for UniProt or Sequence Identity Groups). A so-called carousel or slideshow component enables exploration of individual Group members (Figure 1B). Each slide in the carousel furnishes details about a specific Group member, encompassing its name, organism of origin, and relevant experimental information, such as resolution and molecular weight. Additionally, each slide provides direct links to the Group member’s Structure Summary Page.

**FIGURE 2:**
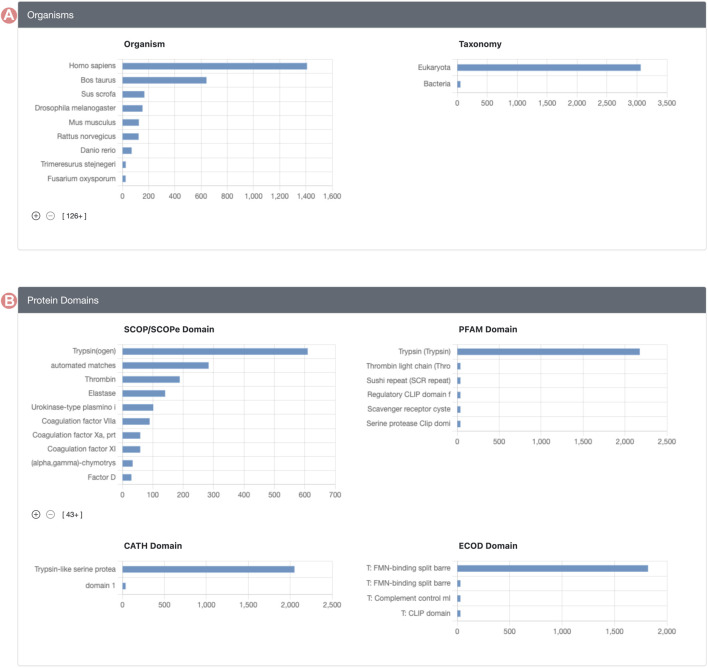
Serine protease Sequence Identity Group Summary Page features. Group member features are in different histograms organized in different sections. **(A)** Organisms section includes source organism histogram (right) and taxonomy (left). **(B)** Group protein domain section includes the distribution of the domains found within Group member proteins, including, SCOP, PFAM, CATH, and ECOD domain classification databases.

Group member properties are presented in the form of histograms or distributions, organized into distinct sections (Figure 2). Each histogram portrays the distribution of a specific property for Group members. For example, the SCOP (Andreeva et al., 2020; Chandonia et al., 2021) domain histogram shows the count of Group members associated with each domain class in the SCOP classification. Supplementary Table S1, in Supplementary Material, provides a comprehensive enumeration of all Group member properties displayed on the GSP.

**FIGURE 3:**
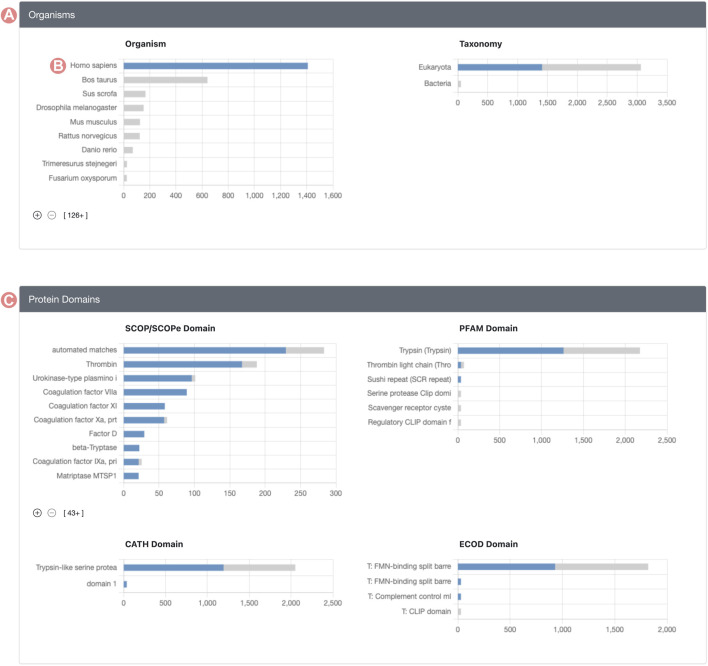
Serine protease Sequence Identity Group Summary Page features. Group histograms are interactive and allow the selection of subgroups. **(A)** Organisms section displays a stacked distribution of *Homo sapiens* Group members in blue color and the rest of elements in gray color. **(B)** The *H*. *sapiens* subset of the Group can be selected by clicking on the histogram bar. **(C)** Group protein domain section displays the stacked distribution of domains for *H*. *sapiens* in blue color and the rest of Group members in grey color.

Histogram chart bars are interactive, allowing users to click on them to explore and define subsets of the original Group. Upon selecting a histogram bar, every other chart adjusts to show the distribution of members relating to the selected property (Figure 3). For example, if a user clicks the bar representing *H. sapiens* in the organism distribution, all histograms are adjusted to display the distribution of members identified as *Homo sapiens*. This updated visualization represents the distribution of Group member properties in two stacked colors: blue sections of the histogram denote Group members meeting the selected criteria, whereas grey segments highlight those that do not. This method offers a streamlined approach to contrasting the distribution of the chosen property *versus* the entire membership of the Group.

### 2.3 Group sequence pages

Group Sequence Pages are accessible for UniProt and Sequence Identity Groups *via* the “Group Sequence” tab (Figure 1A). These pages offer 1D sequence information of the Group members divided across three different tabs.

1. Sequence Alignments: Displays a graphical representation of the multiple sequence alignment (MSA) of Group members.
2. Structural Features: Displays a histogram of Group member structural features mapped onto amino acid sequence alignment positions.
3. Binding Sites: Distribution of the protein-ligand binding sites mapped onto the alignment positions.

**FIGURE 4:**
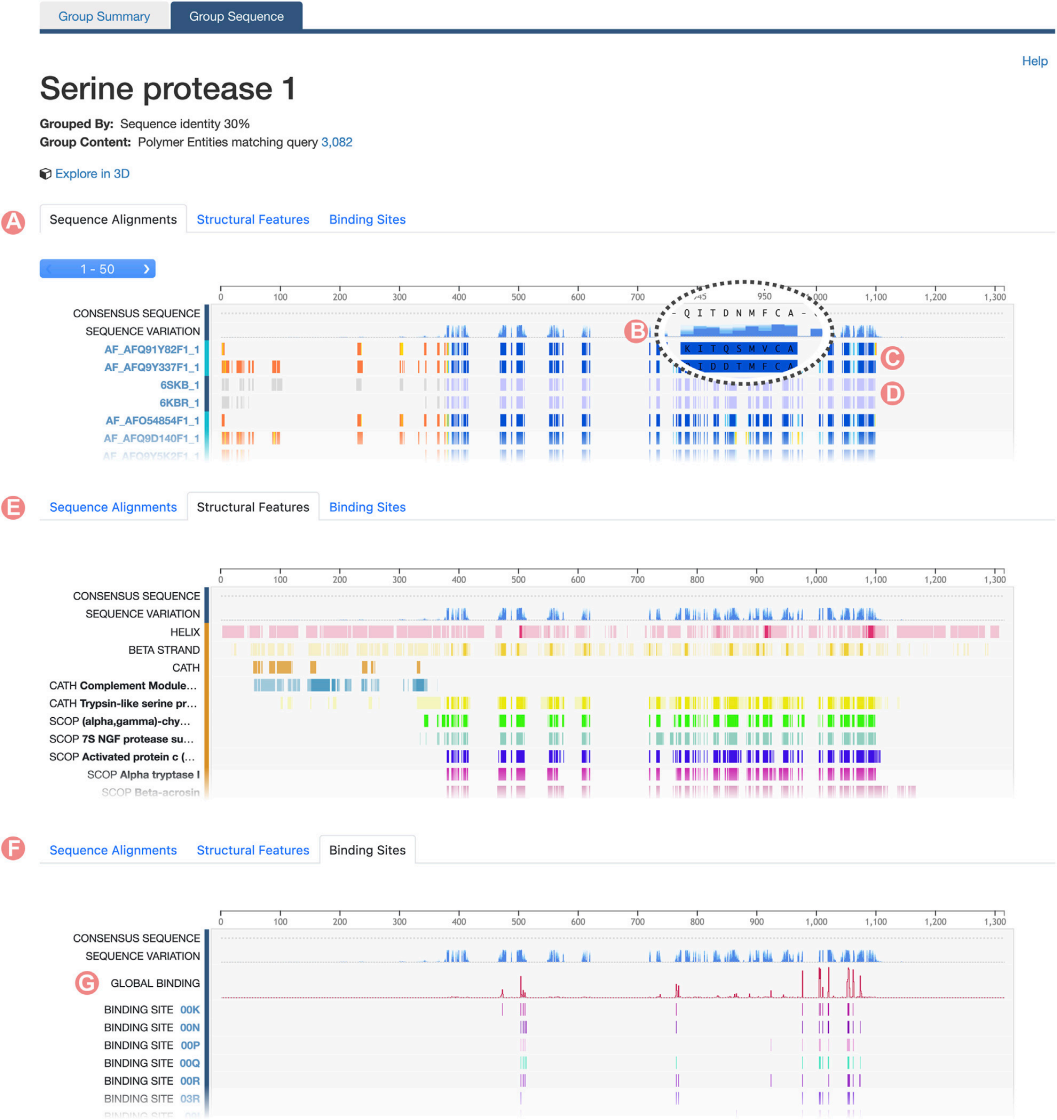
Serine protease Sequence Identity Group Sequence Page. The Group Sequence Pages display sequence positional information for Group members. **(A)** The Alignments tab displays the multiple sequence alignment of Group members. **(B)** The Consensus Sequence and Sequence Variation provides a summary of the amino acid types in the aligned positions. **(C)** Computed structure models aligned regions are displayed based on their local prediction scores (pLDDT). **(D)** Experimentally-determined structures aligned regions are displayed in grey for unmodelled residues and blue for modeled ones. **(E)** Structural features frequency observed in the aligned positions, including, secondary structure and protein domains. **(F)** Ligand binding site frequency observed at the aligned positions. **(G)** Number of interacting ligands for each position.

**FIGURE 5:**
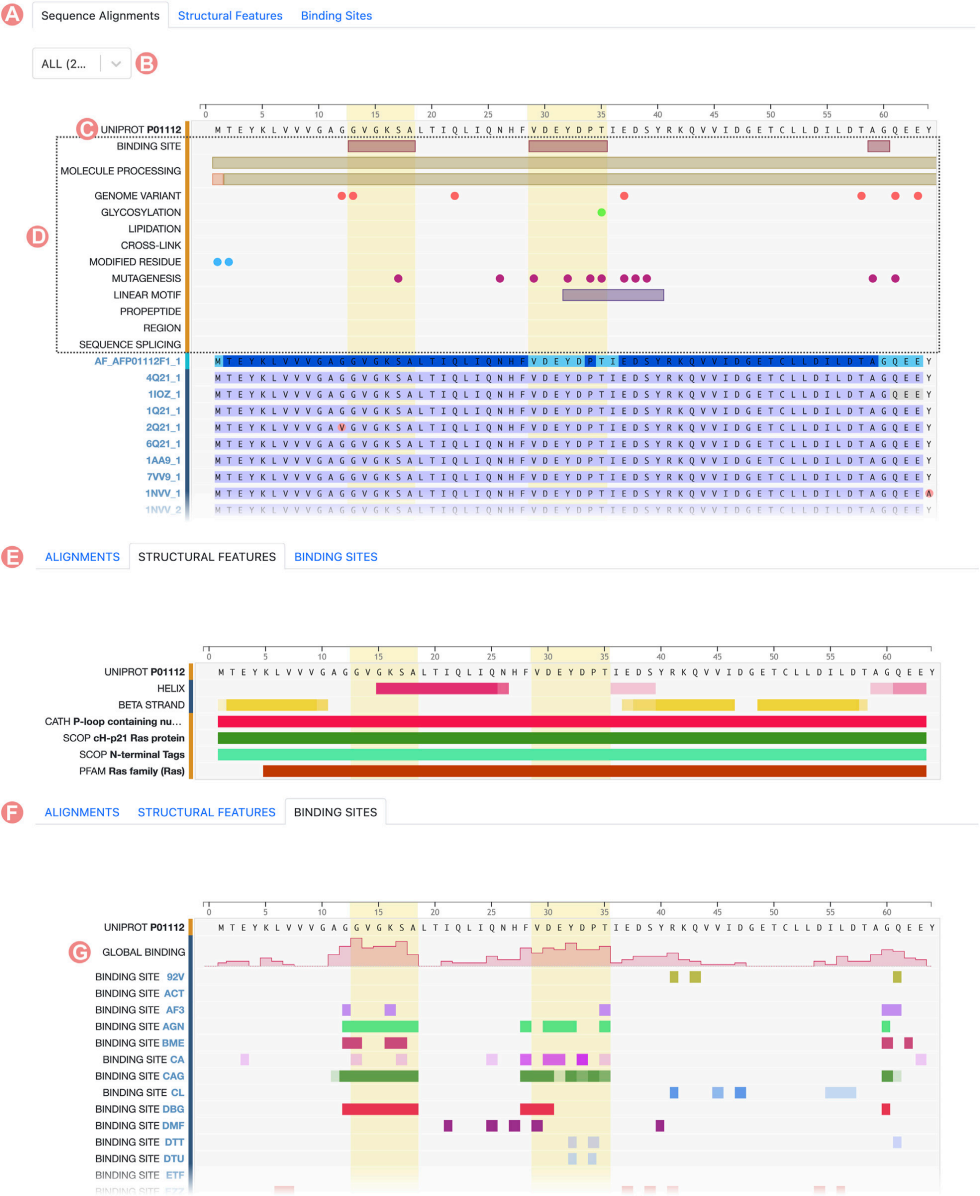
HRas UniProt Group Sequence Page. The Group Sequence Pages display sequence positional information of the Group members. **(A)** The Alignments tab displays UniProt information and the multiple sequence alignment of Group members. **(B)** The select button can be used to display positional features of specific Group members. **(C)** Reference sequence defined as the UniProt sequence. All Group members are aligned to this sequence. **(D)** Positional features sourced from UniProt. **(E)** Structural features frequency observed in the aligned positions, including, secondary structure and protein domains. **(F)** Ligand binding site frequency observed at the aligned positions. **(G)** Number of interacting ligands for each position.

Group Sequence Page content is connected with the information provided on the Group Summary Page. When users select a specific property to inspect a subset of the initial Group, the Group Sequence Page updates to display data pertinent to that subset. Group sequence data are visualized using the RCSB PDB Sequence Annotations Viewer (Table 1; Segura et al., 2020). In this viewer, data are presented in table format. Each table row shows a title and a 1D color-coded diagram representing either aligned regions or specific positional feature histograms (Figure 4). One notable functionality of the viewer is semantic zoom (Figure 4B), empowering users to zoom in and examine individual amino acid sequences in detail. Additionally, users can highlight particular regions within the viewer. Importantly, the displayed region in the viewer, together with any highlights, remains consistent when users switch among different views, such as Sequence Alignment, Structural Features, and Binding Sites (Figure 5).

**TABLE 1:** PDB Group Related Resources. Location of the RCSB PDB Group applications and web services.

Resource name	Location
Sequence Clusters FTP	https://cdn.rcsb.org/resources/sequence/clusters/clusters-by-entity-<threshold>.txt
Search API	https://search.rcsb.org
Data API	https://data.rcsb.org
1D Coordinates API	https://1d-coordinates.rcsb.org
Sequence Annotations Viewer	https://github.com/rcsb/rcsb-saguaro https://github.com/rcsb/rcsb-saguaro-app
1D3D Module	https://github.com/rcsb/rcsb-saguaro-3d

#### 2.3.1 Sequence alignments

By default, the Group Sequence Page viewer presents the MSA of the Group members. However, the information displayed by the Sequence Annotations viewer varies between UniProt and Sequence Identity Groups. For UniProt Groups, the initial track showcases the UniProt reference sequence (Figure 5C), enabling users to discern differences in amino acid composition among the sequences of Group members. Subsequent tracks illustrate sequence positional annotations sourced from the UniProt Knowledge Base (Figure 5D). These annotations offer valuable functional insights, such as post-translational modifications, active sites, or mutations, and they facilitate extrapolation of this information across various Group members.

For Sequence Identity Groups, the MSA is paginated, presenting blocks of 50 tracks at a time. Doing so improves readability when Groups contain hundreds, possibly thousands, of members. Users can navigate between alignment blocks using the pagination button (Figure 4A). The first track of the MSA represents the “Consensus Sequence”. This track represents the most frequently occurring amino acid or gap at each position within the alignment, offering a summary view of the MSA (Figure 4B). Immediately thereafter is the “Sequence Variation” track, which highlights variations in amino acid positions based on relative occurrence. Coloring for this track is gradient-based, transitioning from dark to light blue, with a darker shade indicating lower variation (Figure 4B). In the context of protein families, residues that are highly conserved are often functionally significant.

For both UniProt and Sequence Identity Groups, subsequent tracks display the alignment of individual Group members. Track titles correspond to the PDB Entity or CSM sequence ID and provide links to their respective RCSB.org pages (Figure 4A, Figure 5A). Color coding of the aligned regions depends on the structure-determination methodology. For experimentally-determined structures, regions of the polypeptide chain wherein amino acid atomic 3D coordinates have been defined are displayed in blue, while “unmodelled regions” (for which atomic 3D coordinates are not available) are shown in grey (Figure 4D). For computationally predicted structures, aligned regions are represented by their pLDDT score (Figure 4C). This score is a measure of the estimated accuracy of the predicted amino acid atomic coordinates. Finally, within UniProt Groups, sequence amino acid differences between each Group member and the UniProt reference sequence are indicated with a pink circle on top of the aligned region (Figure 5A).

#### 2.3.2 Structural features

The “Structural Features” tab displays the frequency of different structural features of Group members at certain alignment positions (Figure 4E). In addition, the “Consensus Sequence” and “Sequence Variation” tracks are included to provide summary views of the MSA. Subsequent tracks display the frequency of secondary structure (for amino acid residues occurring with α -helices or β -strands) and protein domain families–defined as CATH (Sillitoe et al., 2021), SCOP (Andreeva et al., 2020; Chandonia et al., 2021), and PFAM (Mistry et al., 2021) domains–across the alignment positions. For example, the “Helix” track shows the frequency of observed α -helical-conformation amino acid residues for each position of the alignment. Frequencies are displayed in a color-gradient alpha channel, where saturated colors indicate a high frequency of occurrence, while low saturation indicates a low occurrence frequency.

#### 2.3.3 Binding sites

Similarly, the RCSB.org “Binding Sites” view provides information on the frequency of protein-ligand interaction sites when binding-ligand amino acid residues of the Group members are mapped onto the sequence alignment (Figure 4F). Again, the “Consensus Sequence” and “Sequence Variation tracks are displayed in the Sequence Alignment viewer as summaries of the MSA. A new track, named “Global Binding”, displays a histogram that measures the number of times each amino acid residue position in the MSA has been observed to interact with a ligand (Figure 4G). Subsequent tracks present the positional binding site frequency for various small molecules known to interact with individual Group members. In this scenario, frequencies are calculated relative to the number of members that interact with a particular ligand. For example, GTP binding site frequencies are computed relative to the number of Group members that interact with GTP. Doing so avoids attribution of near zero frequencies for ligands underrepresented within the Group as a whole.

### 2.4 Group sequence alignments in 3D

**FIGURE 6:**
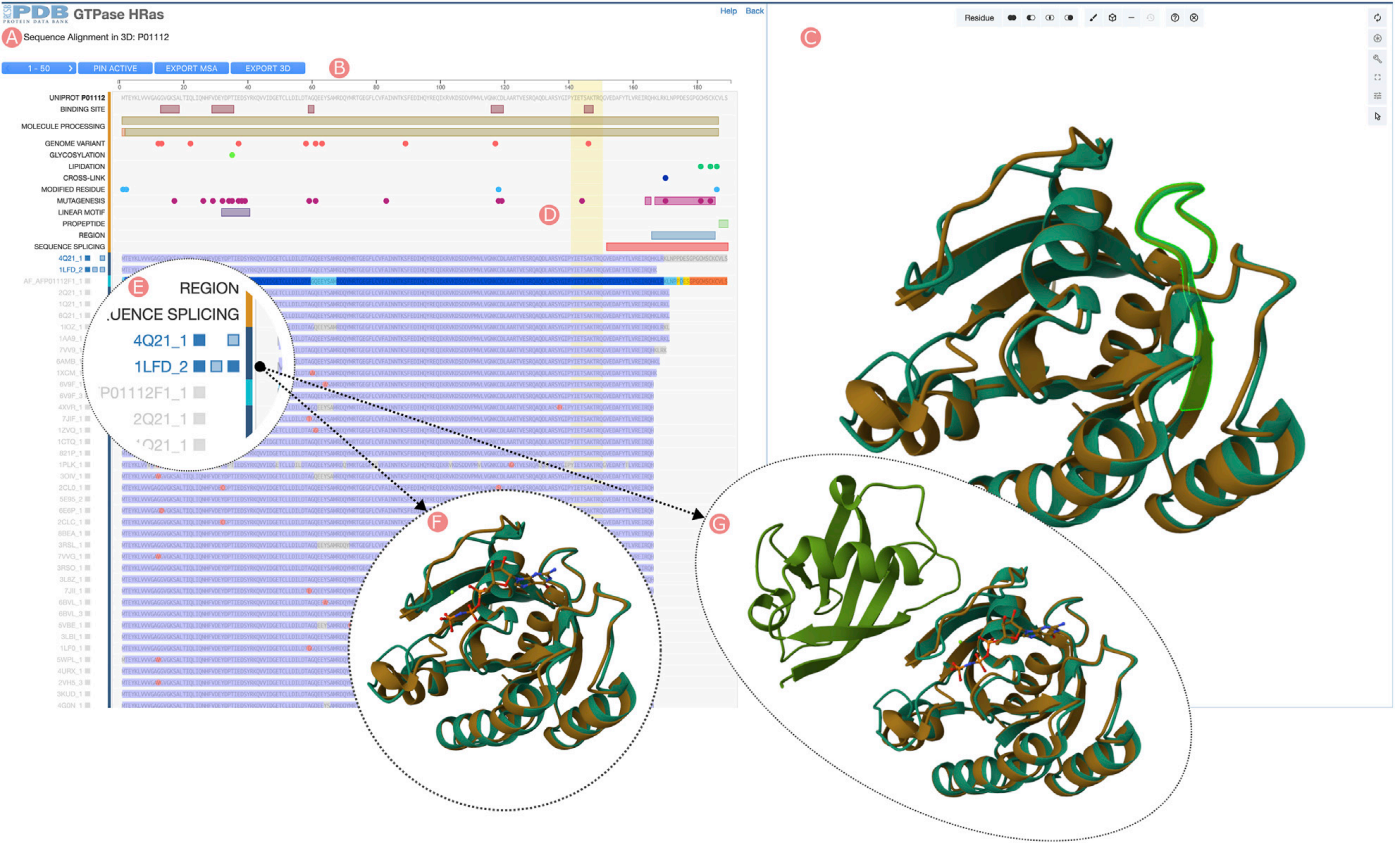
HRas UniProt Group Sequence Alignments in 3D tool. Visualization of the Group member multiple sequence alignment (MSA) and selected 3D atomic coordinates. **(A)** Sequence visualization of UniProt information and the Group members’ MSA. **(B)** Tools menu, including browsing the different MSA pages (50 Group members are displayed), moving to the MSA top selected Group members, downloading aligned sequences of selected Group members, and downloading 3D structures of the selected Group members. **(C)** Protein structure visualization of the selected Group members. **(D)** Selecting sequence regions will highlight in green color the structural position of their residues in the 3D viewer. **(E)** Group members can be selected by clicking on the track titles. Selected Group member titles are displayed in blue color. When active, three checkboxes allow toggling visibility of aligned protein chains, other polymeric chains found in the PDB structure, and ligands. **(F)** The third checkbox next to the track title can be used to display the ligands found in the PDB structure. **(G)** Other polymeric chains can be displayed by clicking on the second checkbox next to the track title.

The Group “Sequence Alignments in 3D” tool is accessible from both the Group Summary Pages Carousel and the Group Sequence Pages by clicking the “Sequence Alignments in 3D” link (Figure 1F). This application uses the RCSB PDB 1D3D Module (Segura et al., 2022) to integrate two visualization tools: the Sequence Annotations Viewer, which showcases the Group MSA, and the Mol* 3D viewer, presenting the structures of Group members (Figure 6). While Group MSA visualization mirrors the method described in Section 3.2.1, a notable distinction is that clicking on track titles triggers loading of Group member 3D structures into the Mol* viewer. Concurrently, three small blue buttons appear on the right side of these titles, enabling users to toggle the visibility of other polymeric chains and small molecules that may be present in a given PDB structure (Figure 6E). Whenever a new set of 3D atomic coordinates is loaded into the Mol* viewer, RCSB.org software performs a rigid-body 3D alignment to superimpose it with a designated reference (*i.e*., the first PDB structure or CSM to be loaded). 3D alignments are informed by the Group MSA amino acid relationships between the reference and the newly loaded protein structure (Figures 6A–C). The “Sequence Alignments in 3D” tool facilitates the comparison of Group members’ structures and display potential interactions between chains and ligands across different PDB structure and CSMs by selecting which chains or ligands are visible. (Figures 6F, G). Additionally, 1D and 3D views are bidirectionally interactive; selecting a region in the MSA highlights the corresponding residues in the 3D view and vice versa (Figure 6D). The application also allows downloading of rendered 3D views and Group member MSAs.

### 2.5 Accessing group pages

**FIGURE 7:**
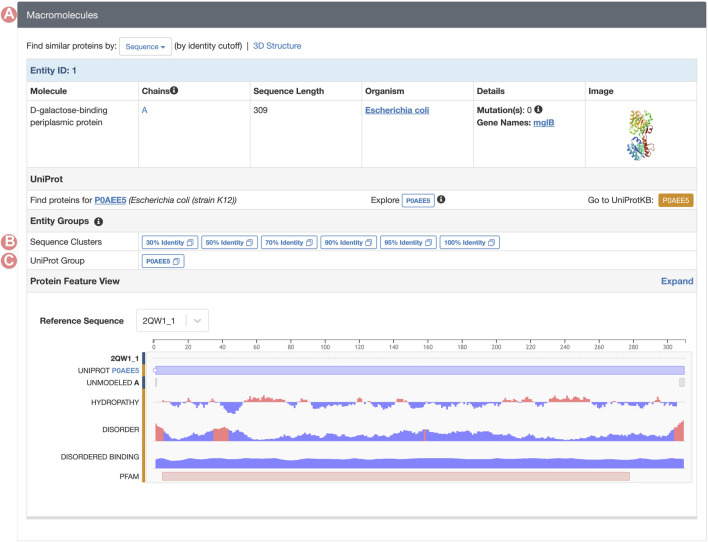
Macromolecules section of the PDB structure 2qw1 Structure Summary Page. **(A)** The macromolecules section can be found on all PDB structure pages. This section contains a description of all PDB Entities in the requested entry. **(B)** Links to the Sequence Identity Groups containing the PDB Entity. **(C)** Link to the UniProt Group associated with the PDB Entity.

GSPs serve as gateways to all Group-related resources on RCSB.org. The web portal offers two distinct pathways to access these pages. First, all RCSB.org Structure Summary Pages, which serve as the entry points for specific PDB structures and CSMs, link each of their proteins (polymeric PDB Entities) to the corresponding UniProt and Sequence Identity GSP Groups (Figures 7B, C). These connections support efficient exploration of structures with analogous properties and analyses within broader contexts (*e.g*., protein structures that share a certain level of sequence similarity). Second, any search result can be grouped through a convenient user interface so that the user can switch from the standard view (each search result item is an individual structure) to a Group display (each search result item is a Group) or to a Group representatives display (each search result item is a representative structure from each Group).

**FIGURE 8:**
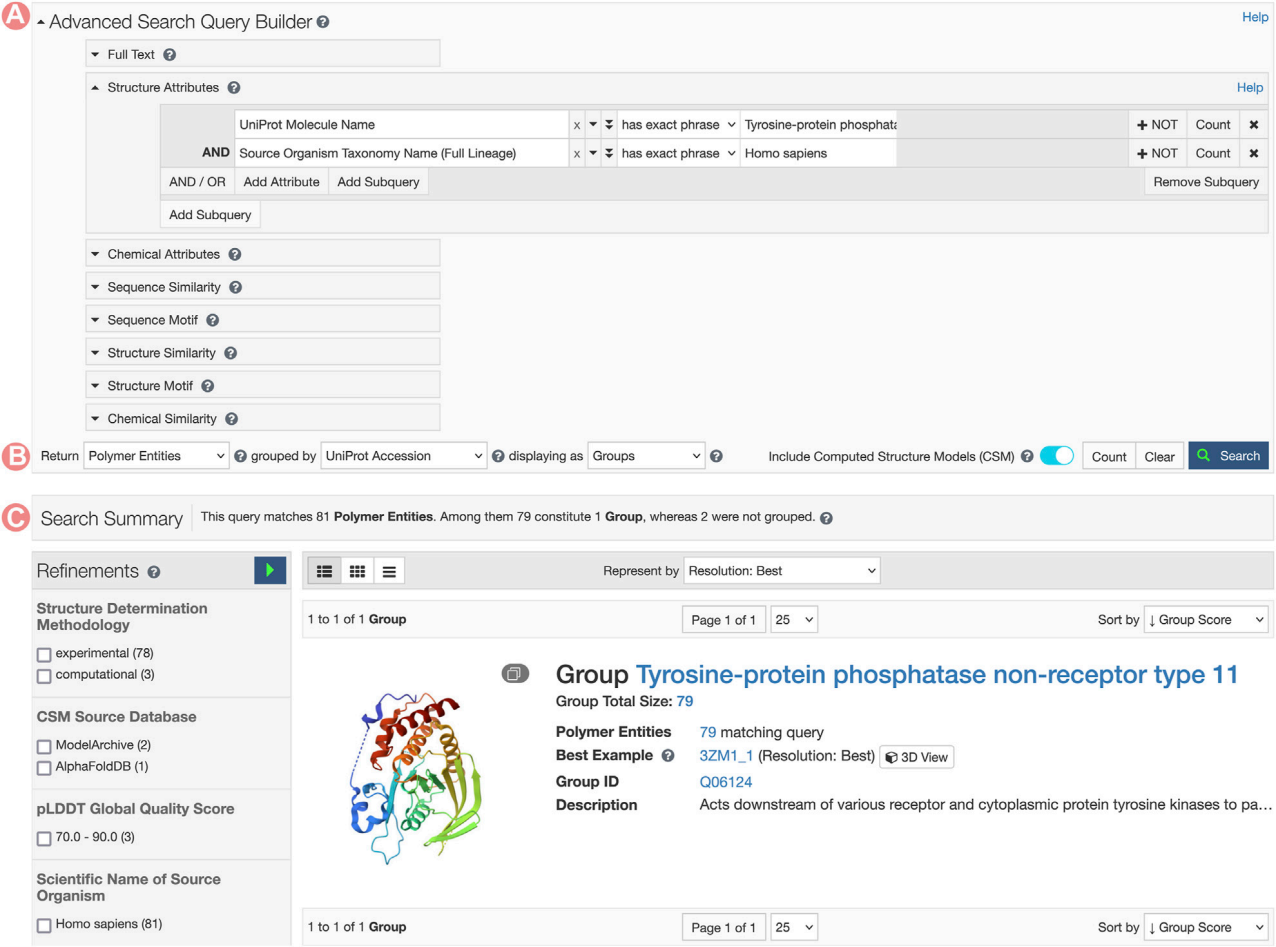
Advanced Search request for Tyrosine-protein phosphatase non-receptor type 11 related Group. **(A)** The Advanced Search Query Builder combines different search strategies to build complex queries. In this example, we combined two Structure Attributes, UniProt Molecular Name, and Source Organism Taxonomy Name, to search for all PDB structures related to the human Tyrosine-protein phosphatase non-receptor type 11. **(B)** The Advanced Search can request different object types. Here, we request UniProt Groups of PDB Entities. **(C)** Search response list. In this case, a single item matches the search conditions.

Available grouping strategies depend on the search result type. Main search result types can be configured to request Structures (PDB structures or CSMs), Polymeric PDB Entities (unique polymer chains within a PDB structure), and Assemblies (specific 3D arrangement of polymeric entities within a PDB structure). For searches yielding PDB structures, results can be organized by Deposition Groups. Polymeric PDB Entities can be grouped based on their UniProt ID or sequence identity clustering (see Section 2.1 and Figure 8). These groupings can be employed to summarize vast collections of structures, streamlining navigation through resultant Groups or Group representatives. When search results are presented as Groups, each item provides an overview of the Group feature, capturing details such as Group name, the number of Group members satisfying the search parameters, the diversity of organisms present in the Group, domain families, and enzymes. When GSPs are accessed from the Advanced Search Application, Groups only contain those members that match the search query. This feature allows users to leverage all advanced search tools in order to build complex queries and explore specific subsets of Groups.

### 2.6 RCSB.org sequence-based clustering

Sequence-based clustering is updated weekly across all PDB protein sequences and incorporated CSMs. Weekly updates ensure that clustering solutions incorporate any new PDB structures released that week and any CSMs newly incorporated at RCSB.org during the previous 7 days. Clustering solutions are generated independently for various thresholds of sequence identity: 30%, 50%, 70%, 90%, 95%, and 100%. Sequence identity clusters are computed using MMseqs2 (Steinegger and Söding, 2017) in a parallel pipeline that utilizes the Luigi workflow engine (https://luigi.readthedocs.io/). Once all clustering solutions are finalized, each cluster receives a unique identifier and is stored in the RCSB.org Data Warehouse (Rose, Y. et al., 2021).

Precomputed sequence clusters play important roles in organizing search results based on sequence identity. When the RCSB.org Advanced Search application yields a list of polymeric PDB or CSM Entities (protein sequences), they can be grouped based on their sequence cluster ID membership. This approach facilitates real-time clustering for any search request. When search results are grouped, result items can either be displayed as Group representatives or as Group descriptions (see Section 2.5).

### 2.7 RCSB PDB APIs

The RCSB.org web portal sources information *via* publicly-available APIs from nearly 50 trusted external data resources. API endpoints facilitate searching, clustering, and data delivery in the RCSB.org web portal. New schema definitions and service endpoints were required to support the various Group functionalities.

#### 2.7.1 Data API

The Data API serves as the gateway to the RCSB PDB Data Warehouse (DW). The DW is a JSON (JavaScript Object Notation) document-based store containing core information for the RCSB.org web portal and related APIs. Documents within the DW are validated using JSON schemas (json-schema.org), which describe the hierarchy of macromolecular structures archived in the PDB. Every week, an ETL (Extract, Transform, and Load) pipeline updates the DW, incorporating newly released PDB structures (Rose, Y. et al., 2021). The Data service is implemented as a lightweight application that mirrors the DW collections and schema types, offering both GraphQL and REST-based APIs.

With introduction of Groups, new endpoints were implemented to access Group-related data in DW. Specifically, the “group_provenance” endpoint enables users to retrieve metadata pertaining to specific grouping methodologies. For example, Code S1, in Supplementary Material, illustrates the request made to gather information about the method used to group PDB structures and CSMs based on sequence similarity. The response will include the aggregation granularity (PDB structure or PDB Entity), the name of the clustering software, description, and version. The “polymer_entity_group” query can be used to fetch information on PDB Entity Groups (see Code S2 in Supplementary Material). The service returns the Group description, a list of the Group members, and, when available, Group member ranking. Group member ranking defines the order of the elements within their Group. Currently, only UniProt grouping provides ranking criteria based on the sequence coverage between UniProt and PDB Entity sequences. The “entry_group” request is homologous to the PDB Entity-based queries to collect information for Groups aggregating PDB structures. Additionally, Group member alignments are available for both UniProt and Sequence Identity Groups. Aligned regions are stored at the level of polymer Entity documents. This information can be accessed using the “core_polymer” API endpoint (see Code S3 in Supplementary Material).

#### 2.7.2 Search API

The RCSB PDB Search API (see Table 1) serves as the primary entry point for all search operations (Rose, Y. et al., 2021). This service consolidates various independent search services, encompassing text and structured attributes, sequence similarity, sequence motifs, structure similarity, structure motifs, and chemical similarity. The Search API merges and combines results from these underlying search services.

Furthermore, searches can be tailored across the different levels of structural data granularity. For example, searches can be configured to request PDB structures, polymeric PDB Entities, or PDB Assemblies. The search service can also furnish statistical data that describes the distribution of structural information based on the annotated attributes. For example, it can provide the distribution of PDB structures released over the years or the histogram of organisms found in a sequence identity Group made up of PDB structures and CSMs. This API is the driving force behind Advanced Search at RCSB.org and stands as the primary statistical data source for constructing histograms in the Group Summary Pages (refer to Section 2.2).

Introduction of results grouping in searches led to implementation of new configuration options in the search request. Code S4, in Supplementary Material, exemplifies a search request that clusters results at 100% of sequence identity. To group search results, users can specify the clustering configuration within the “request_options” attribute. The “group_by” configuration allows users to select the aggregation strategy (“aggregation_method”). The aggregation options include grouping by Deposition, Sequence Identity, or UniProt ID Groups. The Group configuration also enables users to select how Groups are ranked or sorted (“ranking_criteria_type”) and, when applicable, determine cluster parameters such as the sequence identity threshold. Additionally, search results can be fetched either as Group definitions or as Group representatives (“group_by_return_type”).

#### 2.7.3 1D coordinates API

The 1D Coordinate Service (see Table 2) provides pairwise alignment information for various RCSB.org sequence resources and facilitates mapping of positional features between them (Segura et al., 2020). This service provides alignments for both amino acids and nucleotides across the sequence resources integrated therein, including polymeric PDB Entities and CSMs, UniProt sequences, and NCBI genome and protein sequences. Additionally, it amalgamates positional features derived from PDB structures, CSMs, and UniProt annotations, allowing mapping onto any of the integrated sequence references (PDB Entities, CSMs, UniProt and NCBI). For example, the service can map protein-ligand binding sites found in PDB structures to corresponding proteome and genome positions. This service is pivotal for displaying the sequence annotation views on RCSB.org web pages.

The 1D Coordinate Service also exposes a GraphQL API, allowing users to specify the content they need from the resource. To facilitate visualization of sequence annotation views on Group Sequence Pages, we developed two new endpoints. First, the “group_alignment” request allows users to collect MSA information for a specific Group (see Code S5 in Supplementary Material). This function returns a list of Group members together with aligned regions occurring within the Group MSA. Additionally, the service can include the information needed to construct an alignment consensus sequence and the positional variation of the Group MSA (Section 2.3.1). Request parameters encompass the Group reference type (options include “matching_uniprot_accession” or “sequence_identity”), the Group identifier, and an optional parameter that filters specific Group members. Filtering enables coordination of content between Group Summary Pages and Group Sequence Pages when users explore subsets by selecting specific properties of the group members (*e.g*., protein sequences for a specific organism; Section 2.3). Second, “group_annotations” gathers positional features of Group members (Code S5 in Supplementary Material). This API call fetches all structural features and UniProt annotations of Group members, mapping them onto the Group MSA. Optionally, the service supports merging of positional features of Group members to present a histogram detailing positional occurrences of annotations over the MSA positions. This functionality enhances the Group Sequence Page, showcasing structural features within the Sequence Annotation Viewer (Section 2.3.2). Parameters for the “group_annotations” request include the Group reference type, Group identifier, annotation sources, annotation filters, and a histogram flag. This flag is a Boolean, determining whether annotations will be presented as histograms.

## 3 Results

### 3.1 Exploring 3D structures of protein-tyrosine phosphatase

This example illustrates how the Group “Sequence Alignments in 3D” tool can be used to efficiently explore and compare sequence annotations and 3D structural data among Group members. We analyze the structural differences in human Tyrosine-protein phosphatase non-receptor type 11 (PTPN11) resulting from various amino acid mutations. PTPN11 is a hydrolase enzyme that regulates myriad cell signaling processes, including cell growth, differentiation, and the cell cycle. Several mutations in its amino acid sequence have been linked to different diseases, such as Noonan syndrome (Tartaglia et al., 2001), acute myeloid leukemia (Alfayez et al., 2021; Stasik et al., 2021), myelodysplastic syndromes (Tartaglia et al., 2001; 2003), plus other malignancies (LaRochelle et al., 2016).

RCSB.org users can access the PTPN11 Group Summary Page through the Advanced Search application by building a query that combines the ‘UniProt Molecule Name’ and ‘Source Organism Taxonomy Name’ from the Structure Attributes field. Additionally, it is important to configure the return options to fetch PDB entities and group them by UniProt ID. This query will retrieve a single Group of structures related to the PTPN11 protein (Figure 8). From the PTPN11 Group Summary Page, the “Sequence Alignments in 3D” tool can be accessed by clicking on the slideshow link (Figure 1F).

**FIGURE 9:**
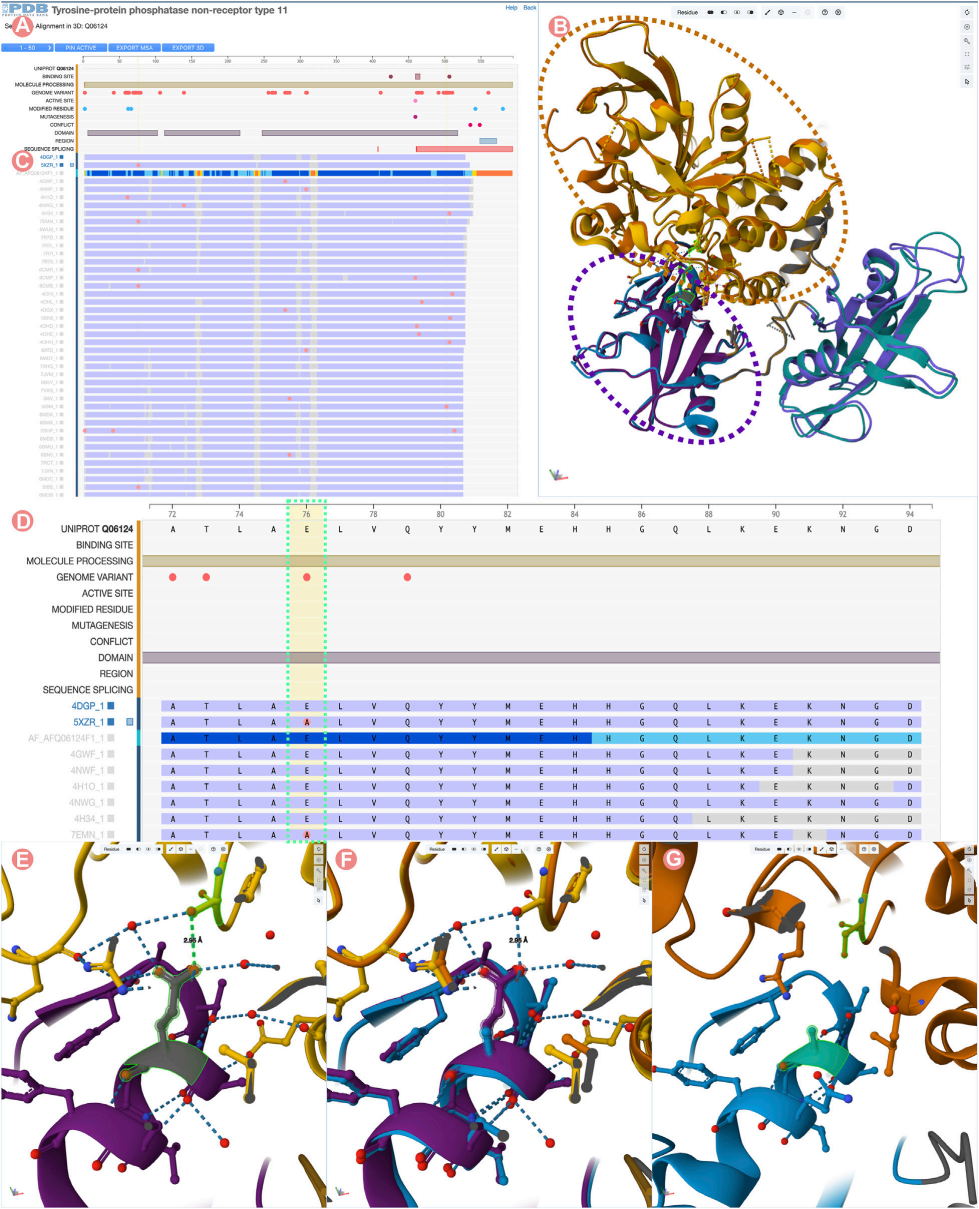
Comparison of Tyrosine-protein phosphatase non-receptor type 11 wild type (PTPN11) and E76A mutant. **(A)** UniProt positional features and multiple sequence alignment view of the PTPN11 UniProt Group. **(B)** 3D structural superposition of PTPN11 wild type and the E76A mutant form of the protein. N-terminal SH2 domains are surrounded by a purple dashed line, and catalytic PTP domains by an orange dashed line. **(C)** Selected Group proteins: wild type (PDB ID 4dgp) and E76A mutant (PDB ID 5xzr). **(D)** Zoom into the Group MSA. Amino acid variations with respect to the UniProt sequence are displayed as pink circles. **(E)** Wildtype E76-S502 hydrogen bond highlighted in green. **(F)** 3D superposition of the wildtype and E76A mutant around E76-S502 interaction. **(G)** Disruption of the E76-S502 interaction in E76A mutant protein. Highlighted in green E76A and S502 residues.

The “Sequence Alignments in 3D” tool will display the Group MSA and 3D structural information for Group members in two different panels (Figures 9A, B). On the left side, the Sequence Annotations Viewer showcases the aligned regions of the Group members with the UniProt sequence and annotations displayed on top. On the right side, the 3D viewer displays 3D atomic coordinates of selected Group members. The Group MSA encompasses both PDB structures and CSMs. Aligned sections are shaded according to pLDDT values. In contrast, aligned sequences of PDB structures are represented in two colors: blue for amino acid residues with experimentally-determined 3D atomic coordinates and grey where there is no structural information. Moreover, differences in amino acid sequences when compared to the UniProt reference sequence are highlighted with pink circles appearing above the aligned regions (Figure 9D). Such 1D depictions of positional features simplify the process of identifying and selecting structures of interest from a Group.

In this example, we compare the PDB structure of the PTPN11 wild-type with one bearing the E76A mutation. Amino acid residue E76 is located within the N-terminal SH2 domain of PTPN11 and forms a critical interdomain hydrogen bond with S502 located in the catalytic domain PTP (Figure 9E). This interaction plays a crucial role in inhibiting substrate access to the active site. Its disruption leads to weakened interactions between the SH2 and catalytic domains, destabilizing the autoinhibited conformation (Xie et al., 2017). Various diseases are associated with disruption of the E76-S502 interaction (Kondoh et al., 2003; Noda et al., 2016). In particular, the E76A mutation is frequently found in malignant cells isolated from individuals diagnosed with acute myeloid leukemia (Tartaglia et al., 2003).

To visualize/depict the E76A mutation, we used the Sequence Annotation viewer to identify and select a PDB structure of wild-type PTPN11 (PDB ID 4dgp) and a PDB structure of comparable quality for the E76A mutant form of PTPN11 (PDB ID 5xzr). Figures 9C, D show how the 1D viewer represents the amino acid sequences of both structures highlighting with a pink circle the mutated residue number 76. Displaying mutations in this way enables identification and selection of appropriate 3D structures. Figures 9B,E,F show a 3D superposition of PDB IDs 4dgp and 5xzr, with SH2 and PTP domains shown in different colors and the E76-S502 interaction highlighted in green. Distinct PTP11N domains can be easily identified from the UniProt annotations. When zooming into the E76-S502 interaction (Figures 9E–G), it is clearly evident how the mutation E76A disrupts the interaction between Sh2 and PTP domains that stabilizes the autoinhibited form of the enzyme (Figure 9G).

In this example, we have analyzed the E76A mutation, but following similar steps, other mutations of PTPN11 can be easily identified and compared with the wild-type protein at the level of sequence and in 3D in atomic detail.

### 3.2 Analysis of a periplasmic protein-ligand antagonist

This use case demonstrates how the Group tools can be employed to compare proteins that undergo conformational changes and explore how interactions with ligands may influence 3D structure. We analyze a receptor antagonist of the *Escherichia coli* D-galactose/methyl-galactoside binding periplasmic protein (GGBP). GGBP is a periplasmic binding receptor (PBP) that mediates various physiologic processes, including transport, chemotaxis, and quorum signaling. In the absence of ligands, PBP receptors exist in both open and closed conformations (Shilton, B.H. et al., 1996). This family of receptors recognizes a variety of small molecule ligands and transitions from an open to a closed state when certain ligands bind. In the closed conformation, these proteins can be recognized by other membrane receptors (Hollenstein et al., 2007). Thus, ligand binding acts as a switch to toggle between the inactive form (open conformation) and the active form (closed conformation).

GGBP is a sugar-binding protein composed of two globular domains, with the sugar-binding site located in a cleft between the domains. Upon binding of D-Glucose or D-Galactose, the domains come together forming a stable closed conformation, which is the active state of the GGBP receptor (Borrok et al., 2007). Small molecules that prevent this conformational change inactivate the receptor. For example, 3-O-methyl-D-glucose (3-OMe-Glc) is a GGBP antagonist that blocks the glucose/galactose binding and prevents formation of the closed, active conformation (Jack Borrok et al., 2009). Here, we will use the RCSB.org Group tools to understand the molecular mechanism that prevents GGBP from transitioning to an active state at the atomic level in 3D.

**FIGURE 10:**
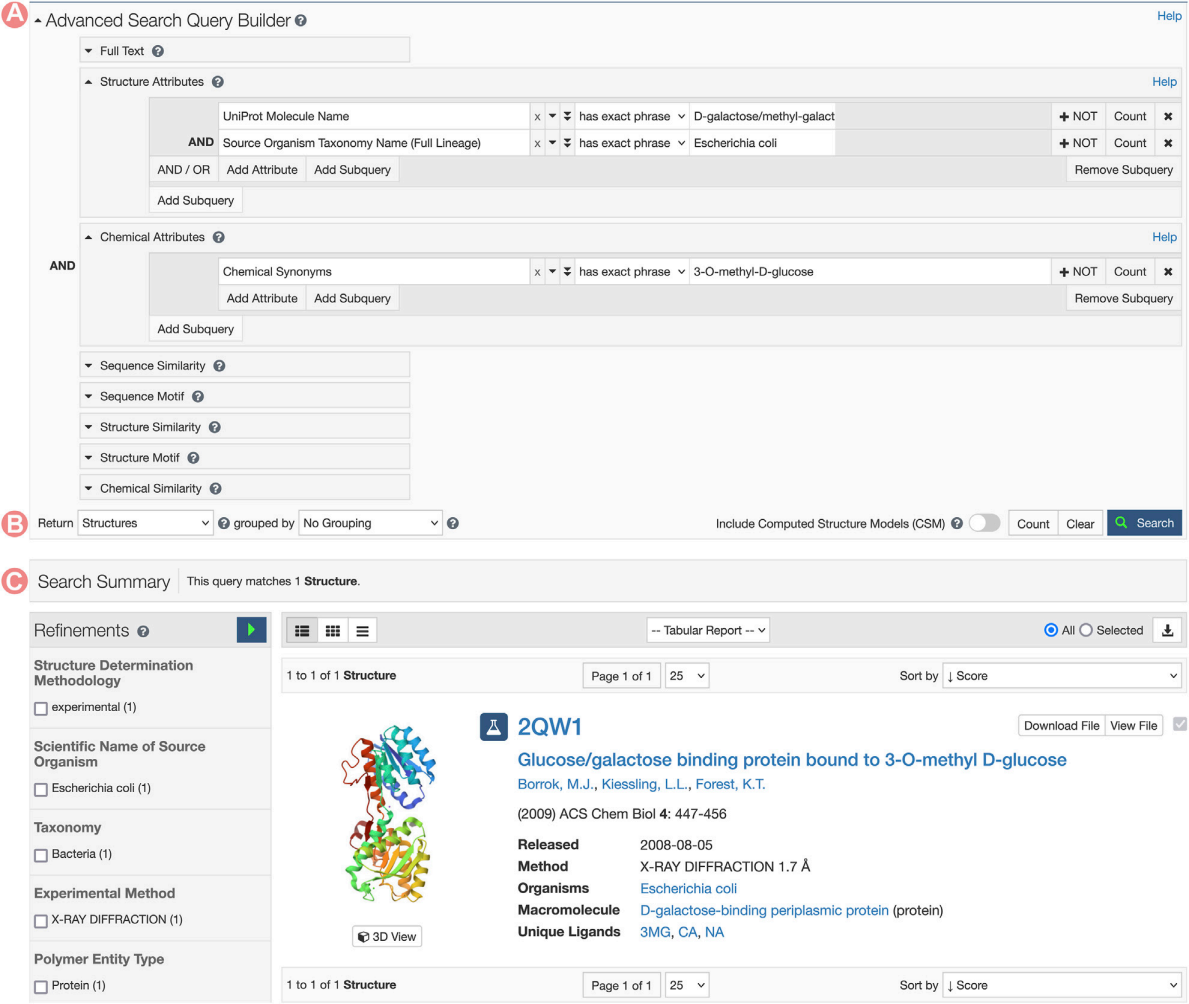
RCSB.org Advanced Search request for the Escherichia coli D-galactose/methyl-galactoside binding periplasmic protein (GGBP) interacting with the 3-O-methyl-D-glucose ligand. **(A)** The Advanced Search Query Builder can combine different search services to request data. In this example, we use two Structure Attributes: UniProt Molecular Name, and Source Organism Taxonomy Name, combined with the Chemical Attribute Chemical Synonyms to search for all PDB structures related to GGBP and containing the 3-O-methyl-D-glucose ligand. **(B)** Advanced Search configuration for requesting different object types. PDB structures are requested. **(C)** A unique entry matches the search conditions.

GGBP Group Summary Pages can be accessed from any of the corresponding individual Structure Summary Pages, wherein the Macromolecules section provides links to all Group pages that cluster related PDB structures and CSMs (Figures 7B, C). In this example, we aim to analyze the 3-O-methyl-D-glucose (3-OMe-Glc) antagonist of GGBP. To identify a PDB structure containing this ligand, we can utilize the RCSB.org Advanced Search function to build a query using appropriate structure and chemical attributes (Figure 10). This request will return a unique PDB structure (PDB ID 2qw1) that includes a single GGBP protein. From its Structure Summary Page, we can access all Group pages related to PDB ID 2qw1 (Figures 7B, C). To analyze and compare this entry with other PDB structures that share the same amino acid sequence as the GGBP in 2qw1, we can select the sequence cluster Group with 100% sequence identity.

**FIGURE 11:**
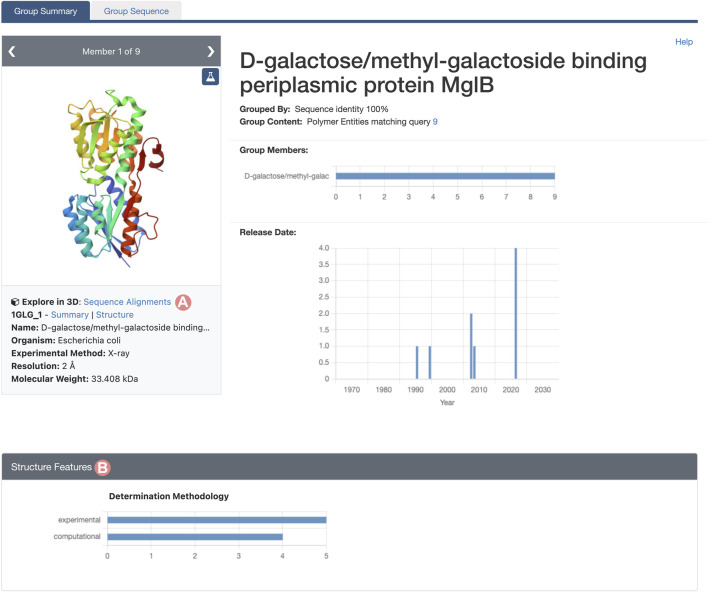
Group Summary Page for the *Escherichia coli* D-galactose/methyl-galactoside binding periplasmic protein. Header and Structure Features section of the Group page. **(A)** Link to the “Sequence Alignments in 3D” tool for the Group. **(B)** The Determination Methodology histogram shows that the Group contains five experimentally-determined structures and four CSMs.

**FIGURE 12:**
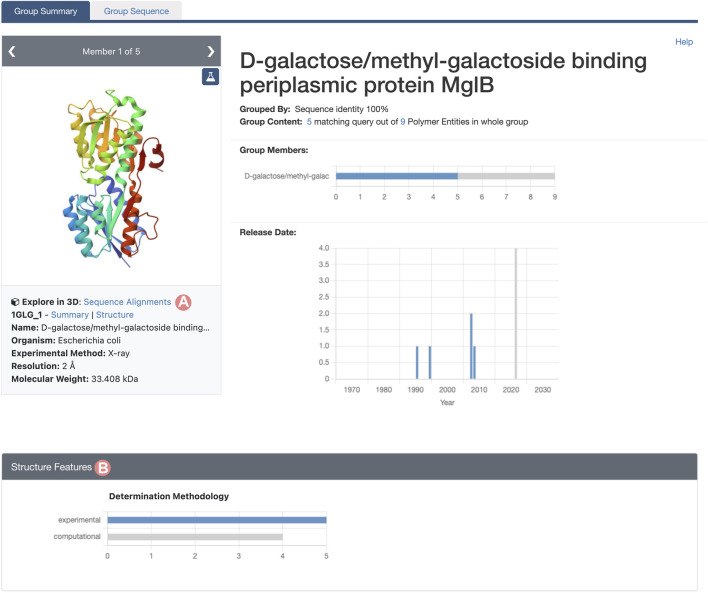
Group Summary Page for the *Escherichia coli* D-galactose/methyl-galactoside binding periplasmic protein. Header and Structure Features section displays the distribution of experimental structures in blue and computed structure models in grey. **(A)** Link to the “Sequence Alignments in 3D” tool for the Group. In this case, the “Sequence Alignments in 3D” tool will display experimental structures only. **(B)** The Determination Methodology histogram has been used to select the experimentally-determined structures of Group members by clicking on the histogram bar.

As outlined in Section 2.2, the Group Summary Page will display the distribution of various attributes using multiple histograms. The “determination methodology” distribution reveals that this cluster contains five experimentally-determined PDB structures and four CSMs (Figure 11B). In this case, if we wish to compare experimental structures under different conditions, we can utilize the “determination methodology” histogram to filter out the CSMs by clicking on the “experimental” bar of the chart. As a result, all charts will be updated to highlight the distribution of the Group members that are PDB structures (Figure 12). We can then access the “Sequence Alignments in 3D” tool by clicking on the link in the slide deck component (Figure 11A; Figure 12A). Any filters applied to the Group members will be retained; in our context, the “Sequence Alignments in 3D” tool will only display experimentally-determined PDB structures.

**FIGURE 13:**
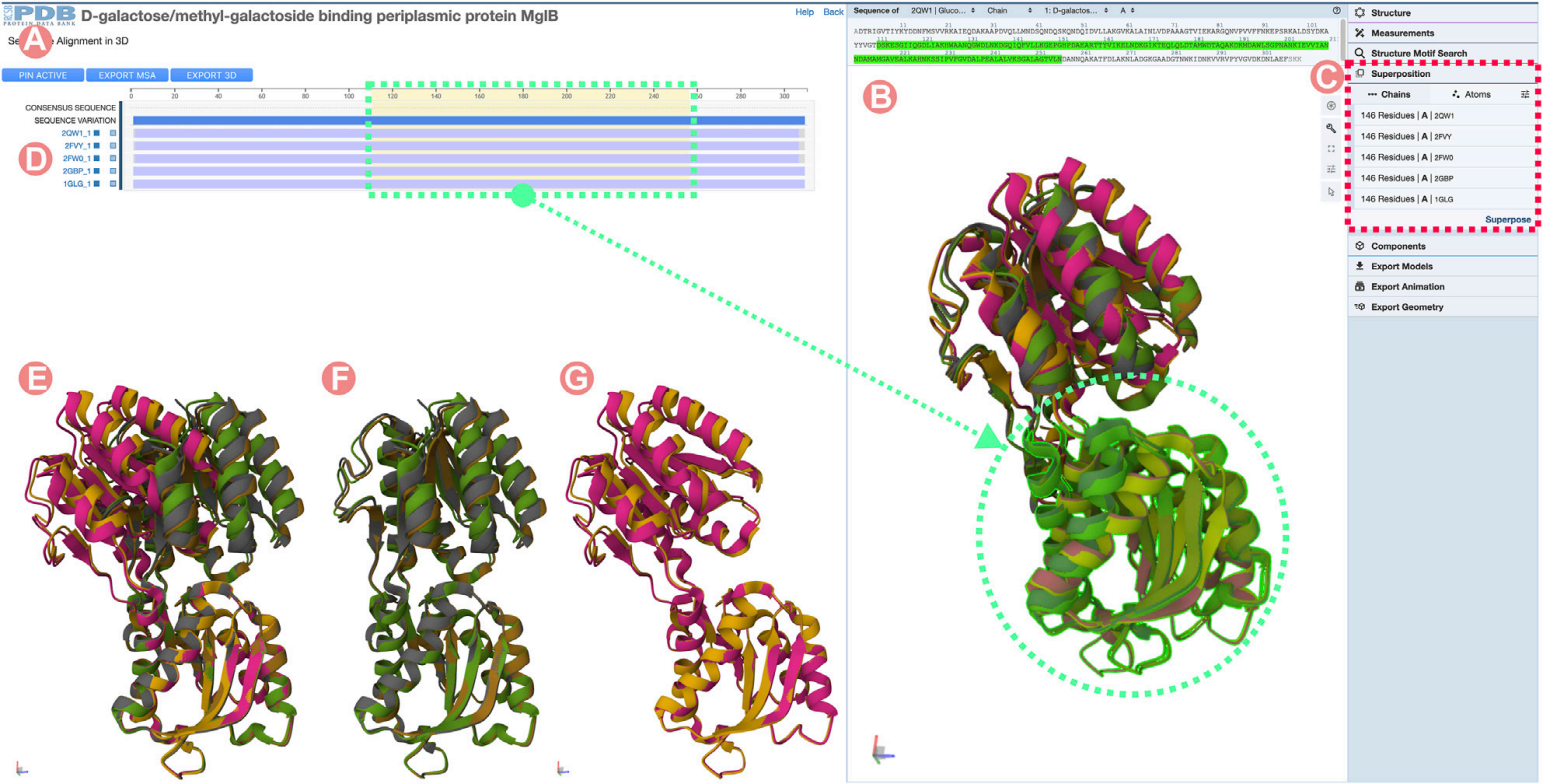
Sequence Alignments in 3D tool for the D-galactose/methyl-galactoside binding periplasmic protein (GGBP) related Group at 100% sequence identity. The GGBP Group includes both active (closed) and inactive (open) conformations of the protein structure. **(A)** Multiple sequence alignment (MSA) of the GGBP proteins. Selected regions on the MSA will select and highlight the 3D residues in the structure viewer for all structures. **(B)** Structure viewer displaying all GGBP structures. When loaded structures are superposed globally, it is challenging to distinguish conformational changes. **(C)** Mol* superposition tool can be used to align the structure of the selected regions. **(D)** MSA track title can be clicked to load individual Group member 3D atomic coordinates. In this example, all Group member atomic coordinates were selected and loaded in the 3D viewer. **(E)** Visualization of the GGBP structures with their C-terminal domain coordinates superposed. This visualization allows us to distinguish between active and inactive conformation. **(F)** Active (close) conformations with their C-terminal domain superposed. **(G)** Inactive (open) conformations with their C-terminal domain superposed.

**FIGURE 14:**
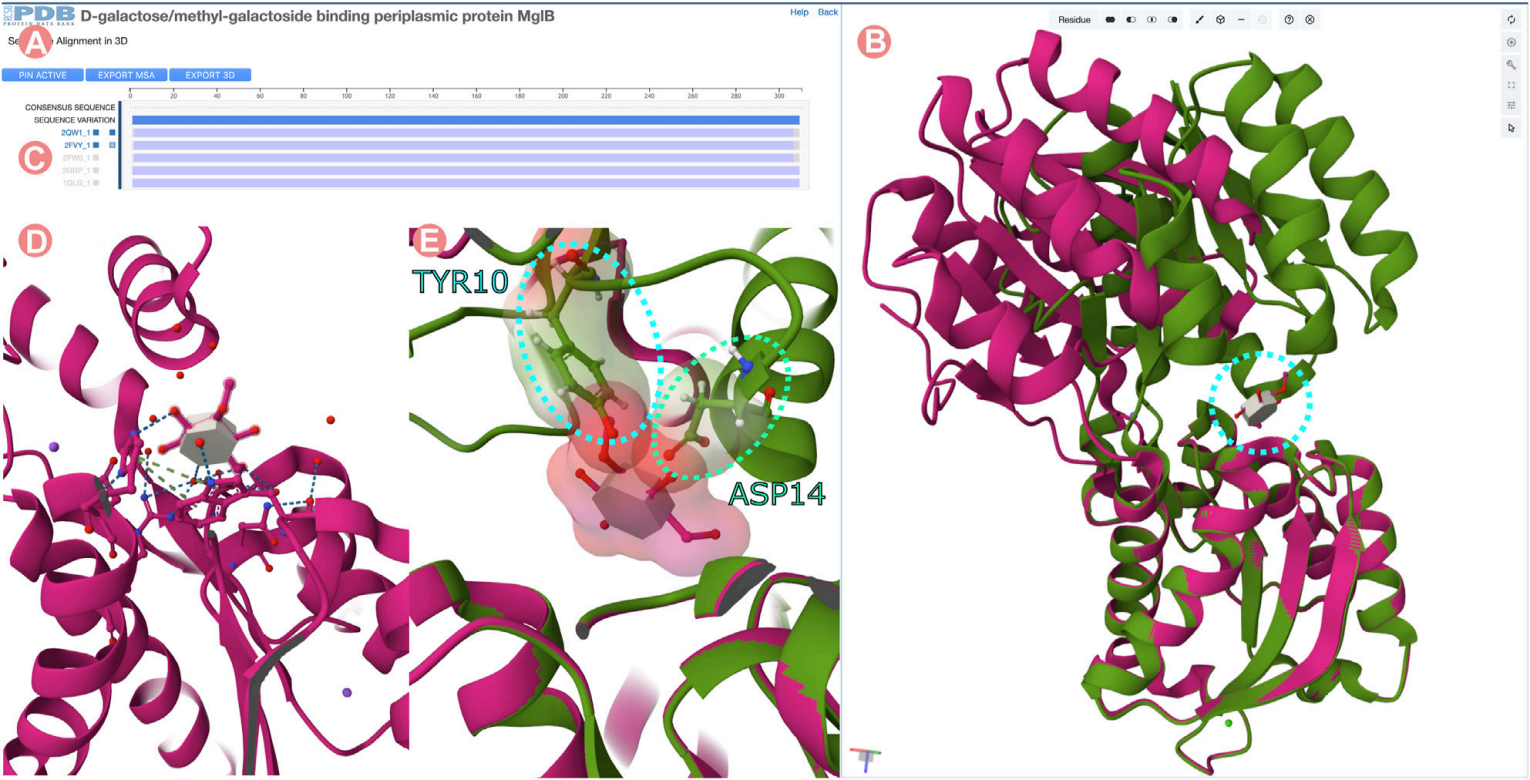
Sequence Alignments in 3D tool for the D-galactose/methyl-galactoside binding periplasmic protein (GGBP) related Group at 100% of sequence identity. “Sequence Alignments in 3D” tool visualizing active and inactivated forms of GGBP protein. **(A)** Multiple sequence alignment displaying all experimentally-determined structures of the GGBP Group. **(B)** 3D structure of inactivated GGBP interacting with the 3-O-methyl-D-glucose ligand (3-OMe-Glc) in magenta color and active conformation in green. C-terminal domains of the structures are superposed. **(C)** The atomic coordinates of Group members can be loaded by clicking on the MSA track titles. In this case, PDB structures 2qw1 and 2fvy were selected. **(D)** 3D visualization of the 3-OMe-Glc ligand interacting with GGBP. The ligand interacts only with the C-terminal domain of the protein. **(E)** Atomic clashes appear between the N-terminal domain of GGBP and the 3-OMe-Glc ligand when the C-terminal domain of both conformations active and inactivated are superposed.

To identify Group members occurring in both open and closed conformations, we can load all the 3D structures by clicking on the Sequence Annotation viewer alignment titles. By default, the 3D structures are superimposed based on the aligned amino acid sequence positions. In cases where alignments cover all sequences, the RMSD will be minimized globally, making it difficult to distinguish between the different conformational states (Figure 13B). To avoid this unwanted outcome, we can superimpose just the C-terminal domains by selecting the region in the Sequence Annotation viewer and using the superposition tool of Mol* (Figure 13C). Figure 13E shows all of the GGBP PDB structures with their C-terminal domains aligned in the 3D. Now, two different domain arrangements are evident: three of them in the closed, active conformation and the other two in the open, inactive state (Figures 13F, G). To continue with the analysis, we will select a PDB structure of the inactivated form of GGBP bound to 3-OMe-Glc ligand (ID 2qw1) and another PDB structure (ID 2fvy) of the GGBP in a closed conformation (Figure 14C).

Figure 14B illustrates the superimposition of the open and closed conformations of GGBP. The structure of the 3-OMe-Glc–GGBP protein-ligand complex provides insights into why the 3-OMe-Glc ligand prevents approximation of the two domains to inactivate GGBP. Superimposition of the C-terminal domains of both structures reveals that upon domain closure, the 3-OMe-Glc ligand would engage in unfavorable steric interactions with certain amino acid residues located in the N-terminal inter-domain cleft. Specifically, the aromatic ring of the GGBP Y10 amino acid residue would make an unfavorable steric clash with the methoxy group of the ligand, and the carboxylate sidechain of D14 would interact unfavorably with the 4-hydroxyl group of the ligand (Figure 14E). Thus, GGBP cannot both bind 3-OMe Glc, and adopt the closed, active conformation.

## 4 Discussion

A new set of tools has been introduced to allow RCSB PDB research-focused web portal RCSB.org users to explore and analyze large collections of protein structures. The primary motivation for developing these new features is to provide ways to remove redundancy within extensive collections of PDB search results and to offer innovative ways of visualizing Groups of related proteins. RCSB.org Advanced Search tools now provide new configuration options to cluster search results based on different properties. When the search results encompass PDB structures, they can be clustered based on Deposition Groups (see Section 2.1). If the search returns polymeric PDB or CSM Entities, they can be grouped according to sequence identity clustering or on the basis of UniProt IDs (see Section 2.1). Additionally, when search results are clustered, users can browse the resulting Groups and explore individual Groups at will. A newly introduced landing page, known as the Group Summary Page, displays the distribution of biological and structural features of Group structures through multiple histograms (see Section 2.2). These pages link to other Group resources to support exploration of sequence and 3D structure information. Group Sequence Pages provide MSAs of Group members and summarize structural features of Group members mapped onto reference sequences within MSAs (see Section 2.3). Finally, the “Sequence Alignments in 3D” tool offers an efficient means of exploring the Group member structures, allowing users to compare differences between sequences and 3D conformations.

## Data Availability

The original contributions presented in the study are included in the article/Supplementary Materials, further inquiries can be directed to the corresponding author.
