# Supplementary material for: RCSB Protein Data Bank: visualizing groups of experimentally determined PDB structures alongside computed structure models of proteins
Source: Front Bioinform. 2023 Dec 4;3:1311287. doi: 10.3389/fbinf.2023.1311287 (PMC10726007; doi:10.3389/fbinf.2023.1311287)
Supplement: Supplementary file 1 [file DataSheet1.PDF]

Supplementary Material

RCSB Protein Data Bank: Visualizing groups of experimentally determined PDB structures alongside computed structure models of proteins

Joan Segura*, Yana Rose, Chunxiao Bi, Jose Duarte, Stephen K. Burley, Sebastian Bittrich

*** Correspondence:** Corresponding Author: joan.segura@rcsb.org

1 Supplementary Data

```
query group_provenance {
  group_provenance(
    group_provenance_id: "provenance_sequence_identity"
  ){
    rcsb_group_aggregation_method {
      type
      method {
        name
        details {
          description
          name
        }
        version
      }
    }
    rcsb_group_provenance_container_identifiers{
      group_provenance_id
    }
  }
}
```

Code S1. Group provenance Data API request. GraphQL request to retrieve information of a group aggregation methodology (link).

```
query polymer_entity_group {
  polymer_entity_group(
      group_id: "P01112"
  ){
    rcsb_id
    rcsb_group_container_identifiers{
      group_provenance_id
      group_id
    }
    rcsb_group_info{
      group_members_granularity
      group_members_count
      group_description
      group_name
    }
    rcsb_group_container_identifiers{
      group_member_ids
    }
    rcsb_group_statistics{
      similarity_cutoff
    }
    rcsb_polymer_entity_group_members_rankings{
      ranking_criteria_type
      group_members{
        member_id
        original_score
      }
    }
  }
}
```

Code S2. Polymer entity group Data API query. GraphQL request to retrieve in formation of a polymer entity group based on UniProt aggregation (link).

```
query polymer_entity_aligned_reions {
  polymer_entity(
    entry_id: "101M"
    entity_id: "1"
  ){
    rcsb_id
    rcsb_polymer_entity_group_membership{
      group_id
      aligned_regions{
        entity_beg_seq_id
        ref_beg_seq_id
        length
      }
    }
  }
}
```

Code S3. Group member sequence alignment region Data API query. GraphQL request to collect the aligned regions of a group member (link).

```json
{
  "query": {
    "type": "group",
    "logical_operator": "and",
    "nodes": [
      {
        "type": "terminal",
        "service": "sequence",
        "parameters": {
          "sequence_type": "protein",
          "value": "MNGTEGPNFYVPFSNKTGVVRSPFEAPQYYLAEPWQFSMLAAYMFLLIMLGFPI",
          "identity_cutoff": 0.3,
          "evalue_cutoff": 0.1
        }
      },
      {
        "type": "terminal",
        "service": "text",
        "parameters": {
          "attribute": "exptl.method",
          "operator": "exact_match",
          "value": "X-RAY DIFFRACTION"
        }
      }
    ]
  },
  "request_options": {
    "group_by": {
      "aggregation_method": "sequence_identity",
      "similarity_cutoff": 100,
      "ranking_criteria_type": {
        "sort_by": "entity_poly.rcsb_sample_sequence_length",
        "direction": "desc"
      }
    },
    "group_by_return_type": "groups"
  },
  "return_type": "polymer_entity"
}
```


Code S4. Search API request. Search request clustering results at 100% of sequence similarity (link).

(A)

```
query group_alignment_request {
  group_alignment(
    group: sequence_identity
    groupId: "1_30"
  ){
    alignment_logo{
      value
      symbol
    }
    target_alignment{
      target_id
      target_sequence
      aligned_regions{
        query_begin
        query_end
        target_begin
        target_end
      }
    }
  }
}
```

(B)

```
query group_annotations_request {
  group_annotations(
    group: sequence_identity
    groupId: "1_30"
    sources: [PDB_ENTITY,UNIPROT]
  ){
    source
    target_id
    features{
      type
      provenance_source
      description
      feature_id
      name
      feature_positions{
        beg_seq_id
        end_seq_id
      }
    }
  }
}
```

Code S5. 1D Coordinates Service query. (A) GraphQL request to retrieve the multiple sequence alignment of a group based on sequence identity clustering (link). (B) GraphQL request to collect the sequence annotations of a group mapped onto the group multiple sequence alignment positions (link).

2 Supplementary Figures and Tables

**Header**	
Group Members	Distribution of protein names of group members
Release Date	Distribution of group structures release date
**Structure Features**	
Determination Methodology	Number of group structures determined by experimental methods and computationally predicted
**Experimental Features**	
Experimental Method	Distribution of experimental techniques used to solve the group structures
Resolution	Distribution of resolution values for experimental structure solved by X-ray crystallography or Cryo-EM
**Organisms**	
Taxonomy	Distribution of group members amog the different domains of life (Eukaryota, Bacteria, Archea, …)
Organism	Histograms of scientific organism names of the group members (*Homo sapiens, Mus musculus, Escherichia coli, …*)
**Protein Domain**	
SCOP Domain	Histogram of protein domains found among the group members based on different protein domain classification resources
CATH Domain	
PFAM Domain	
ECOD Domain	
**Function**	
Enzyme Classification	Histogram of the different enzymes found in the group
GO Biological process	Group members histogram of Gene Onthology terms
GO Molecular Function	
GO Cellular Component	
**Small Molecules**	
Small Molecules	Count of protein-ligands interactions observed among the grop members

Table S1. Group member properties displayed in Group Summary Pages.
